# Prognostic values of the gross volume of metastatic lymph nodes in patients with esophageal squamous cell carcinoma treated with definitive concurrent chemoradiotherapy

**DOI:** 10.3389/fonc.2022.996293

**Published:** 2022-11-10

**Authors:** Yang Li, Yanqi Li, Hui Huang, Zhoubo Guo, Kunning Zhang, Wencheng Zhang, Qingsong Pang, Ping Wang

**Affiliations:** Department of Radiation Oncology, Tianjin Medical University Cancer Institute and Hospital, National Clinical Research Center for Cancer, Key Laboratory of Cancer Prevention and Therapy, Tianjin’s Clinical Research Center for Cancer, Tianjin, China

**Keywords:** esophageal cancer, chemoradiotherapy, gross tumor volume of metastatic lymph nodes, prognosis, stage system

## Abstract

**Purpose:**

We aim to explore whether the gross volume of metastatic lymph nodes (GTVnd) and the gross volume of primary tumor (GTVp) could be prognostic factors for esophageal squamous cell carcinoma (ESCC) patients treated with definitive concurrent chemoradiotherapy (dCCRT).

**Methods:**

We retrospectively analyzed 252 ESCC patients treated with dCCRT in the era of intensity-modulated radiation therapy (IMRT) at our institution. The cut-off value for the GTVnd derived from the restricted cubic splines (RCS) was determined. Univariate and multivariate Cox proportional hazard models were performed to determine the association between GTVnd and prognosis. we performed recursive partitioning analysis (RPA) method using GTVnd to develop a new risk stratification (TGTVndM). Moreover, the linear trend χ2, likelihood ratio χ2, and akaike information criterion (AIC) were used to determine the prognostic value between the TNM and TGTVndM staging systems.

**Results:**

The five-year overall survival (OS) rate was 30.6%, with a median follow-up of 38 months. The cut-off value of GTVnd determined by the RCS was 4.35 cm^3^. GTVnd≥4.35 cm^3^ was an independent and significant negative prognostic factor for OS (HR=1.949, P<0.001), progression free survival (PFS) (HR=1.425, P=0.048), and distance metastasis free survival (DMFS) (HR=2.548, P=0.001). In multivariable analysis, gender, clinical T stage, and GTVnd were independently associated with OS. RPA segregated patients into 3 prognostic groups: high risk (T1-4 GTVnd≥4.35, n=126, III stage), intermediate risk (T4 GTVnd<4.35,n=38,II stage), and low risk(T1-3GTVnd<4.35, n=88, I stage). The 5-year OS(P<0.001), PFS (P=0.002), and DMFS (P=0.001) were significantly worse in high-risk group in comparison with the intermediate and low risk groups. Compared with the TNM staging system, the clinical T stage combined with GTVnd (TGTVndM) had a higher linear trend χ2 (26.38 versus 25.77), higher likelihood ratio χ2 (24.39 versus 20.69), and lower AIC (1255.07 versus 1260.06).

**Conclusions:**

GTVnd may serve as a good prognostic factor in predicting distant metastasis and death for ESCC patients treated with dCCRT. The TGTVndM staging system demonstrated superior accuracy for predicting OS and could serve as a more effective prognostic guidance for unresectable ESCC patients.

## Introduction

Esophageal cancer (EC) is the sixth most common cancer and the fourth most common cause of cancer-related death in China, with 324,422 new cases and 301,135 deaths estimated based on the Global Cancer Incidence, Mortality and Prevalence 2020 (GLOBOCAN https://gco.iarc.fr/). Histologically, esophageal squamous cell carcinoma (ESCC) is the predominant subtype of EC, accounts for nearly 90% of EC worldwide (1, 2). Concurrent chemoradiotherapy has been widely accepted as standard regimen for unresectable locally advanced EC patients according to RTOG-85-01 trial (3). Despite some advances in past decades, the prognosis remains poor with 5-year OS rate about 20% for all stages combined (4). Thus, finding effective prognostic factors enable to identify patients with poor prognosis after therapy and optimally define risk adapted treatment strategies to further improve the survival rate in ESCC patients.

The TNM staging established by the American Joint Committee on Cancer (AJCC) is considered as a useful staging system to predict outcomes of EC patients underwent surgical resection (5, 6). However, TNM staging is a tool based on pathological anatomy. It is difficult to obtain accurate TNM staging for ESCC patient with dCCRT. And survival of ESCC patients receiving dCCRT with the same clinical TNM stage varies widely. Therefore, it is far from accurate and sensitive enough to use only TNM staging to predict the prognosis of these patients. At present, there is still a lack of sufficient indicators to predict the effect of definitive chemoradiotherapy on ESCC patients.

With the advancement of intensity-modulated radiotherapy (IMRT) technique, more and more precise data on volume of primary tumor and metastatic lymph nodes are available to be collected, making it possible to study the influence of volumetric parameters on the outcomes of patients with ESCC. In this study, we propose that the GTVp and GTVnd may have an impact on the survival of patients based on our clinical experience. Therefore, we investigated ESCC patients treated with dCCRT to identify the effect of GTVp and GTVnd on OS, PFS, DMFS, local recurrence-free survival (LRFS), and regional recurrence-free survival (RRFS).

## Patients and methods

### Patients

We performed a retrospective study in patients with pathologically confirmed inoperable ESCC who were treated with dCCRT in the era of IMRT at Tianjin Medical University Cancer Institute and Hospital from 2010 to 2019. The main inclusion criteria were as follows: 1) pathologically confirmed ESCC; 2) Karnofsky performance status (KPS) score ≥70; 3) no distant organ metastasis; 4) no history of a concomitant or previous malignancy; 5) underwent IMRT-based definitive concurrent chemoradiotherapy; and 6) had unresectable EC or refused surgery. A total of 252 patients with complete clinical and treatment information met the above criteria and were selected for analysis. The study was approved by the Ethics Committee of Tianjin Medical University Cancer Institute and Hospital. The patients were not required to sign an informed consent form for this retrospective study.

### Clinical stage

All patients underwent complete evaluations before treatment including detailed medical history, physical examination, upper gastrointestinal radiography, neck, chest and abdominal contrast enhanced computed tomography (CT), endoscopy with biopsy, endoscopic ultrasound, and external ultrasonography of the neck, and more recently fluorodeoxyglucose (FDG)-PET-CT. Patients were re-staged according to the 8^th^ staging system of the AJCC based on all information provided by EUS and CT-scanning and/or FDG-PET-CT scanning according to radiologists and oncologists at our hospital collectively evaluating. Lymph nodes were considered positive mainly on CT images if they were spherical and had a maximal transverse diameter > 10 mm. Visible lymph nodes < 10 mm on CT images were regarded as metastasis positive only if focal 18F-FDG uptake on PET-CT images was obvious compared with normal mediastinal activity.

### Treatment

All ESCC patients received dCCRT and radiotherapy was performed as IMRT. All treatments were planned based on CT simulation planning system with and without contrast and a slice thickness of 2.5 to 5 mm throughout the entire neck, thorax and upper abdominal under shallow breathing. Primary tumor and metastatic lymph nodes>1 cm (≥5 mm in tracheoesophageal groove) were contoured as primary gross tumor volume (GTVp) and gross tumor volume of metastatic lymph nodes (GTVnd) based on both physical examination and all available diagnostic images. The clinical target volume (CTV) was defined as the visible GTVp, GTVnd and subclinical regions at risk for involvement. The planning gross target volume (PGTV) was obtained by adding an isotropic margin of 0.5 cm to the GTVp combined with GTVnd. The planning target volume (PTV) was defined as the CTV plus a 0.5 cm margin in all directions. Two radiation oncologists reviewed all contoured structures to ensure accuracy and consistency. The dosimetric parameters for GTVp and GTVnd were calculated from the Pinnacle system for every patient.

Patients received concurrent chemotherapy with a weekly or three-weekly schedule of paclitaxel and platinum-based drugs. For subsequent consolidation chemotherapy, chemotherapy regimens were selected based on patient age, general physical condition, and physician judgment.

### Endpoints

The end points included the OS, PFS, DMFS, LRFS, and RRFS. We defined OS as the time from the first treatment to the date of death from any cause or the date of last follow-up. The PFS was calculated from the time of first treatment to disease progression, including local recurrence (LR), regional recurrence (RR), and distant metastasis (DM). The DMFS was set for the period from the date of treatment to distant metastasis. LRFS and RRFS were defined as the intervals between the beginning of treatment and the date of recurrence that occurred in the esophageal lumen, and between the beginning of treatment and the date of regional lymph nodes. Patients who did not experience an event of interest were censored at their last follow-up or the date of death.

### Follow-up

Patients were follow-up at least every 3 months during the first 2 years, every 6 months for 2 to 5 years, and then annually thereafter. The imageological examination including chest CT scans, upper gastrointestinal radiography, and neck and upper abdomen ultrasonography were routinely performed at each follow-up study. In addition, physical examinations, routine blood tests and liver and kidney function analyses were also necessary. The results of the re-examination were confirmed by electronic medical records, paper medical documents, and telephone. If the patients died by the time of contact, available family members provided the needed information.

### Statistical analysis

Categorical variables were summarized using frequencies and percentages, and the χ2 test and Fisher’s exact test were used to compare the differences among different groups. Continuous variables were presented as the median values and interquartile range (IQR). Age, KPS, radiation dose and primary tumor length were categorized with the median value as the cut-off. With the utilization of RCS curves, the association between the GTVnd (as a continuous variable) and OS, PFS, DMFS, RRFS were evaluated based on Cox regression model (7). The Kaplan‐Meier method was used to plot the survival curves and the log‐rank test was used to compare the survival distributions. We used univariate and multivariate Cox proportional hazard models to evaluate the influence of different variables on the OS, PFS, DMFS, RRFS, and LRFS. Adjusted hazard ratios (HR) were obtained with corresponding 95% confidence intervals (95% CIs) from the Cox regression analysis. *P* values were calculated using the Cox regression forward-LR model. Additionally, we use the linear trend χ2 test to measure the discriminatory ability and monotonicities, the likelihood ratio χ2 test based on the Cox regression model to the homogeneity, and the AIC to the optimal prognostic stratifications between the two staging systems (8). A higher linear trend χ2 score or likelihood ratio χ2 score and a lower AIC value indicates a better model for predicting prognosis (9). We use the online web server called autoRPA (available at http://rpa.renlab.org) to establish a decision-making tree from survival data based on the RPA algorithm and log-rank test statistics to correctly stratify risk in the target population  (10). All statistical tests were two-sided and P values < 0.05 were considered statistically significant. Analyses were conducted using SPSS v22.0 (IBM SPSS, New York) and the R 4.1.0 (http://www.r-project.org/).

## Results

### Patient characteristics

We identified 252 patients with pathologically confirmed inoperable ESCC treated with dCCRT from 2010 to 2019 in our study, with complete information of GTVp and GTVnd. The demographic characteristics of the ESCC patients are shown in **Table 1**. The age of the patients ranged from 30 to 82 years with a median age of 60 years. Median GTVp and GTVnd were 38.77 cm^3^ (interquartile range (IQR), 24.00-58.02 cm^3^) and 4.34 cm^3^ (IQR, 0.18-11.38 cm^3^), respectively. And the median ratio between the two (GTVnd/GTVp) was 0.11 with an IQR of 0.01-0.30.

**Table 1:** Characteristics of the patients with esophageal squamous cell carcinoma treated with definitive concurrent chemoradiotherapy (n = 252).

Patient characteristics	No of patients(%)
Age	
Median	60
Range	30-82
Gender	
Male	220 (87.3)
Female	32 (12.7)
KPS	
≥90	173 (68.7)
<90	79 (31.3)
Weight loss	
Yes	89 (35.3)
No	163 (64.7)
Pain of chest and back	
Yes	51 (20.2)
No	201 (79.8)
Tumor location	
Cervical	22 (8.7)
Upper thoracic	78 (31.0)
Middle thoracic	93 (36.9)
Lower thoracic	59 (23.4)
Clinical T stage, 8^th^	
T1	9 (3.6)
T2	16 (6.3)
T3	139 (55.2)
T4	88 (34.9)
Clinical N stage, 8th	
N0	59 (23.4)
N1	86 (34.1)
N2	77 (30.6)
N3	30 (11.9)
Clinical TNM stage, 8th	
I	7 (2.8)
II	42 (16.7)
III	102 (40.5)
IV	101 (40.1)
GTVp (cm^3^)	
Median	38.77
IQR	24.00-58.02
GTVnd (cm^3^)	
Median	4.34
IQR	0.18-11.38
GTVnd/GTVp	
Median	0.11
IQR	0.01-0.30
Tumor length, cm	
≤6*	126 (50.0)
>6	126 (50.0)
Radiation dose, Gy	
<54	75 (29.8)
≥54	177 (70.2)
Consolidation chemotherapy	
Yes	106 (42.1)
No	146 (57.9)

*median tumor length.

### Treatment outcomes

Within the median duration of follow-up for the whole patients of 38 months (ranging from 1 to 89 months), we identified 117 (46.4%) patients developing locoregional recurrence (LRR), 62 (24.6%) DM and 132(52.4%) death. The five-year OS, PFS, DMFS, LRFS and RRFS rates for the entire cohort were 30.6%, 28.4%, 58.0%, 46.9%, and 66.7%, respectively.

### Prognostic value of volumetric parameters on survivals

GTVp, GTVnd and GTVnd/GTVp were segregated into two groups according to their median values. Subsequently, we investigated the prognostic role of the volumetric parameters using univariate and multivariate Cox proportional hazard regression. In univariate analysis (**Table 2**), GTVnd ≥4.34 cm^3^ and GTVnd/GTVp ≥0.11 were associated with poorer OS (both P<0.001), PFS (P=0.001; P=0.014; respectively), DMFS (both P<0.001) and RRFS (P=0.023; P=0.031; respectively). Then the variables with statistical significance in univariate analysis(P<0.05) were included in the multivariate analysis and P values were calculated using the Cox regression forward-LR model. Multivariable analyses validated the independent prognostic role of the GTVnd in OS (HR=1.949, P<0.001), PFS (HR=1.425, P=0.048) and DMFS (HR=2.548, P=0.001). In addition, the result demonstrated advanced clinical T stage (P=0.002) and male (P=0.046) were independently associated with shorter OS, shown as **Table 3**.

**Table 2:** Univariate cox proportional hazard regression analysis of prognostic factors in patients with ESCC (n=252).

Variable	OS*P*	PFS*P*	DMFS*P*	RRFS*P*	LRFS*P*
Age (>60 years vs. ≤60 years)	0.796	0.923	0.412	0.116	0.640
Gender	**0.034**	**0.005**	**0.025**	**0.016**	0.084
KPS (≥90 vs. <90)	0.343	**0.043**	**0.048**	0.144	0.105
Weight loss	0.122	0.637	0.510	0.516	0.628
Pain of chest and back	0.973	0.665	0.167	0.602	0.783
Tumor location	**0.010**	**<0.001**	0.620	**0.015**	0.059
Clinical T stage	**0.001**	**0.011**	0.184	0.324	0.103
Clinical N stage	**0.001**	0.341	0.152	0.639	0.923
GTVp (≥38.77 vs.<38.77cm^3^)	0.167	0.496	0.945	0.475	0.480
GTVnd (≥4.34 vs.<4.34cm^3^)	**< 0.001**	**0.001**	**< 0.001**	**0.023**	0.464
GTVnd/GTVp (≥0.11 vs.<0.11)	**< 0.001**	**0.014**	**< 0.001**	**0.031**	0.887
Tumor length (>6cm vs. ≤6cm)	**0.048**	0.290	0.847	0.259	0.342
Radiation dose (≥54Gy vs. <54Gy)	0.242	0.127	0.060	0.093	0.110
Consolidation chemotherapy	0.633	0.189	0.423	0.110	0.478

Bold value means P < 0.05.

**Table 3:** Multivariate cox proportional hazard regression analysis of prognostic factors in patients with ESCC (n=252).

	HR (95%CI)	P
OS		
T stage		0.002
T stage (T2 vs. T1)	2.492 (0.290-21.405)	0.405
T stage (T3 vs. T1)	3.341 (0.472-24.925)	0.223
T stage (T4 vs. T1)	6.134 (0.844-44.550)	0.073
GTVnd (≥4.34 vs.<4.34cm^3^)	1.949 (1.353-2.808)	< 0.001
Gender (male vs. female)	1.939 (1.013-3.712)	0.046
PFS		
Gender (male vs. female)	2.373 (1.242-4.535)	0.009
GTVnd (≥4.34 vs.<4.34cm^3^)	1.425 (1.003-2.024)	0.048
Tumor location		0.001
Tumor location (UT vs. Cervical)	0.274 (0.146-0.514)	< 0.001
Tumor location (MT vs. Cervical)	0.543 (0.310-0.950)	0.032
Tumor location (LT vs. Cervical)	0.475 (0.261-0.8)	0.015
DMFS		
GTVnd (≥4.34 vs.<4.34cm^3^)	2.548 (1.491-4.355)	0.001

UT, Upper thoracic; MT, Middle thoracic; LT, Lower thoracic.

**Figure 1:**
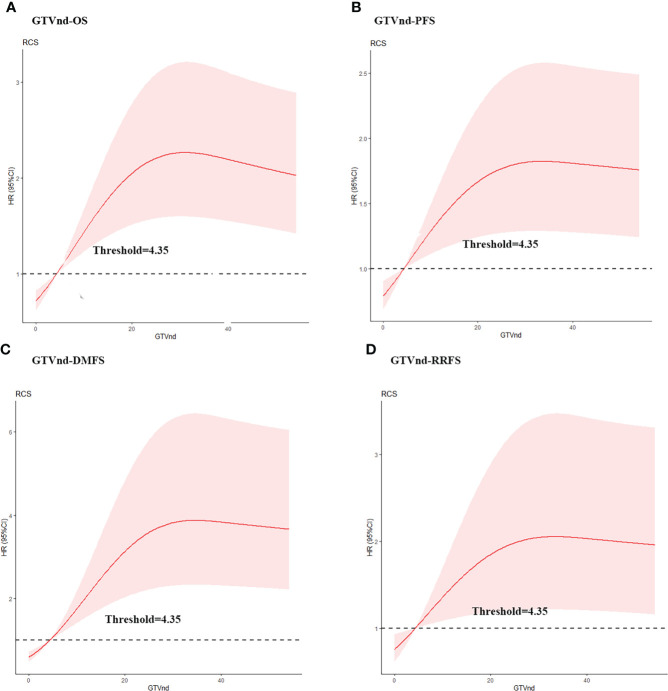
Estimated hazard ratio (HR) (red solid line) with 95% confidence intervals (red dash area) for the association of gross volume of metastatic lymph nodes (GTVnd) with **(A)** overall survival (OS), **(B)** progression free survival (PFS), **(C)** distant metastasis free survival (DMFS), and **(D)** regional recurrence free survival (RRFS) in ESCC patients treated with dCCRT. The risk (in HR) of OS, PFS, DMFS, and RRFS increased along with the augment of GTVnd.

To confirm the optimal GTVnd cutoff value, we used the RCS with 3 knots and OS, PFS, DMFS, and RRFS as endpoint events. The cutoff value determined by RCS was 4.35 cm^3^ in this analysis (**Figure 1**). Multivariable analysis also showed that GTVnd≥4.35 cm^3^ was independent and significant negative prognostic factors for OS, PFS and DMFS.

### Construction of risk grouping using GTVnd by RPA model

**Figure 2:**
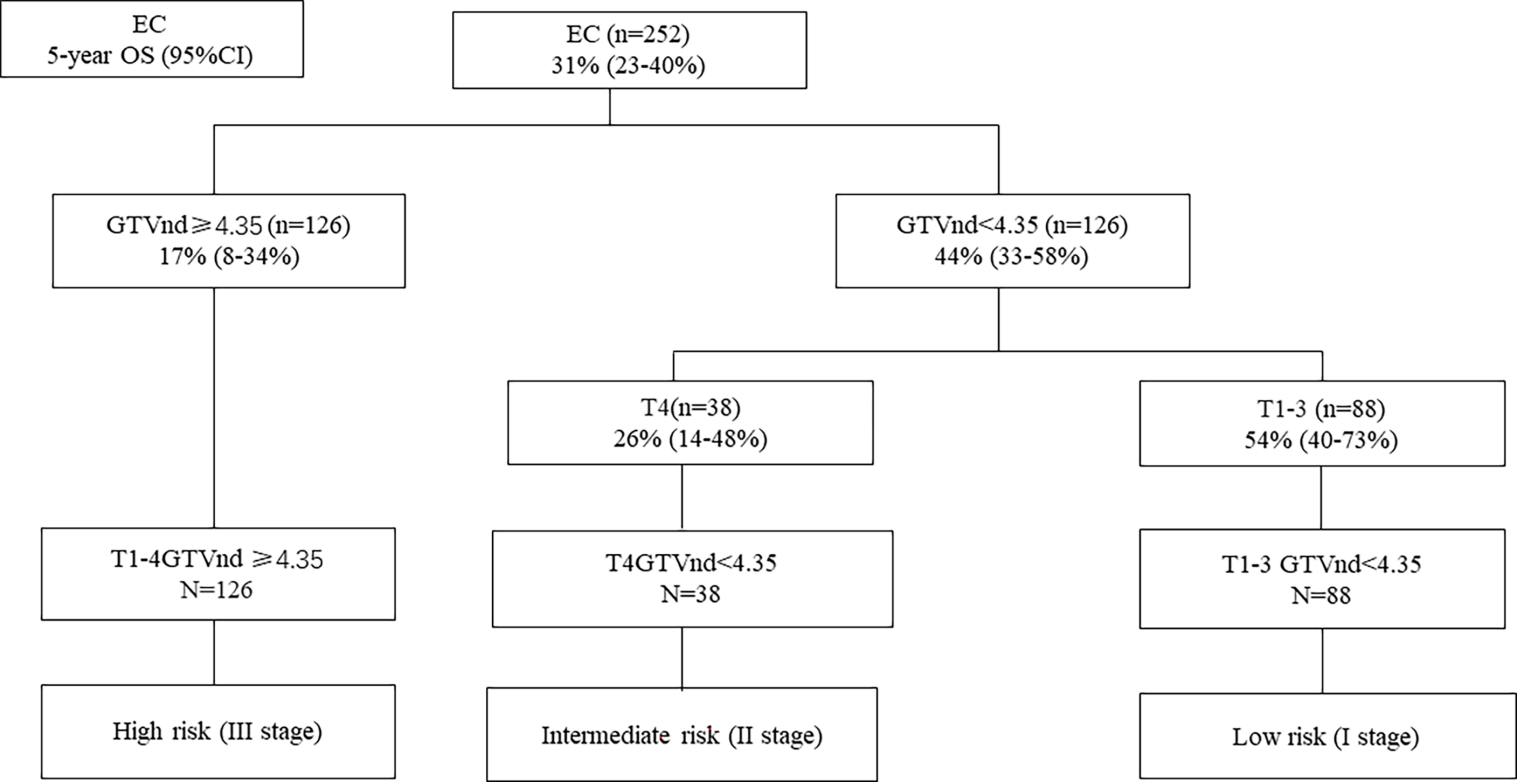
Recursive partitioning analysis (RPA) for OS in ESCC patients based on GTVnd and T stage.

Considering the prognostic value of GTVnd, we then performed RPA algorithm including T stage, GTVnd/GTVp, and gender to develop a new staging. The significant RPA-derived splits were only the T stage and GTVnd (**Figure 2**). The RPA model divided the 252 ESCC patients into the following three groups: high-risk (T1-4 GTVnd≥4.35, n=126, III stage), intermediate-risk (T4 GTVnd<4.35,n=38,II stage), and low-risk(T1-3GTVnd<4.35, n=88, I stage).

Clinical and treatment characteristics of the new risk grouping is shown in **Table 4**. The differences in age, gender, KPS, weight loss, pain of chest and back, tumor length, radiation dose, induction chemotherapy and consolidation chemotherapy were not statistically significant between the two groups (P > 0.05). Compared with those in the high-risk group, patients in the low-risk and intermediate-risk groups whose tumor were more located in the cervical and upper thoracic (48.9%, 52.6% vs.29.3%, P=0.033). And the proportion of N0-1 was higher in low-risk and intermediate-risk groups than that in the high-risk group (87.5%, 81.6% vs. 44.8%, P<0.001).

**Table 4:** Baseline characteristics of patients stratified by new risk stratification.

Characteristic	Low-riskgroup (n = 88)	Intermediate-risk group (n = 38)	High-risk group (n = 126)	*P*
Age				0.751
≤60	44 (50.0%)	19 (50.0%)	69 (54.8%)	
>60	44 (50.0%)	19 (50.0%)	57 (45.2%)	
Gender				0.165
Male	76 (86.4%)	30 (78.9%)	114 (90.5%)	
Female	12 (13.6%)	8 (21.1%)	12 (9.5%)	
KPS				0.083
≥90	53 (60.2%)	26 (68.4%)	94 (74.6%)	
<90	35 (39.8%)	12 (31.6%)	32 (25.4%)	
Weight loss				0.127
Yes	27 (30.7%)	10 (26.3%)	52 (41.3%)	
No	61 (69.3%)	28 (73.7%)	74 (58.7%)	
Pain of chest and back				0.441
Yes	14 (15.9%)	8 (21.1%)	29 (23.0%)	
No	74 (84.1%)	30 (78.9%)	97 (77.0%)	
Tumor location				0.033
Cervical	7 (8.0%)	4 (10.5%)	11 (8.7%)	
Upper thoracic	36 (40.9%)	16 (42.1%)	26 (20.6%)	
Middle thoracic	28 (31.8%)	12 (31.6%)	53 (42.1%)	
Lower thoracic	17 (19.3%)	6 (15.8%)	36 (28.6%)	
Clinical T stage, 8th				<0.001
T1	7 (8.0%)	0 (0.0%)	2 (1.6%)	
T2	12 (13.6%)	0 (0.0%)	4 (3.2%)	
T3	69 (78.4%)	0 (0.0%)	70 (55.6%)	
T4	0 (0.0%)	38 (100.0%)	50 (39.7%)	
Clinical N stage, 8th				<0.001
N0	37 (42.0%)	21 (55.3%)	1 (0.8%)	
N1	40 (45.5%)	10 (26.3%)	36 (28.6%)	
N2	9 (10.2%)	3 (7.9%)	65 (51.6%)	
N3	2 (2.3%)	4 (10.5%)	24 (19.0%)	
GTVnd				<0.001
≥4.35	0 (0.0%)	0 (0.0%)	126 (100.0%)	
<4.35	88 (100.0%)	38 (100.0%)	0 (0.0%)	
Tumor length, cm				0.211
≤6	49 (55.7%)	21 (55.3%)	56 (44.4%)	
>6	39 (44.3%)	17 (44.7%)	70 (55.6%)	
Radiation dose, Gy				0.397
<54	26 (29.5%)	8 (21.1%)	41 (32.5%)	
≥54	62 (70.5%)	30 (78.9%)	85 (67.5%)	
Consolidation chemotherapy				0.174
Yes	33 (37.5%)	21 (55.3%)	52 (41.3%)	
No	55 (62.5%)	17 (44.7%)	74 (58.7%)	

### The prognostic significance of the risk group

**Figure 3:**
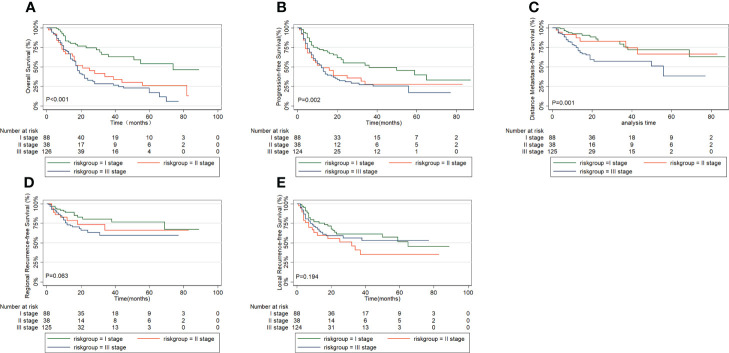
Comparison of survival outcomes in prognostic grouping by recursive partition analysis: **(A)** overall survival, **(B)** progression free survival, **(C)** distant metastasis free survival, **(D)** regional recurrence free survival, **(E)** local recurrence free survival.

Then, we performed the Kaplan-Meier analysis to compare OS, PFS, DMFS, RRFS, and LRFS between the three groups derived by RPA (**Figure 3**). We found highly significant differences in OS among the three groups (P < 0.001; **Figure 3A**), with corresponding 5-year OS rates of 17.0% for high-risk group, 26.3% for intermediate-risk group, and 54.0% for low-risk group. The 5-year PFS rates of high-risk group, intermediate-risk group and low-risk group were 16.6%, 27.6% and 39.8%, respectively. By the log–rank test, there were significant differences in PFS among the three groups (P=0.002; **Figure 3B**). And the 5-year DMFS rates of 37.8% for patients with high-risk group and 72.0% for those with low-risk group showed significant differences (P =0.001; **Figure 3C**). Though a prognostic analysis demonstrated the 5-year RRFS rate in the high-risk group (59.1%) was lower than that of the intermediate-risk group (66.0%) and low-risk group (76.6%), the difference was not significant (P=0.063; **Figure 3D**). No significant difference in LRFS rate was observed among the three groups (P=0.194; **Figure 3E**).

### Comparison of the performance of the new risk grouping and TNM staging system

**Figure 4:**
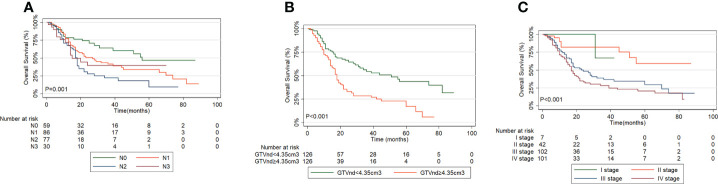
Overall Survival curves for ESCC patients treated with dCCRT according to: **(A)** N stage, **(B)** GTVnd, **(C)** TNM stage system.

The performance between the new risk grouping (TGTVndM) and TNM staging systems assessed by linear trend χ2, likelihood ratio χ2, and the AIC is presented in **Table 5**. The TGTVndM staging system demonstrated higher linear trend χ2 (26.38 versus 25.77), high likelihood ratio χ2(24.39 versus 20.69), and lower AIC (1255.07 versus 1260.06) compared with the TNM stage, indicating the optimum prognostic stratification in predicting the survival of ESCC patients treated with dCCRT and then we compare the overall survival for ESCC patients treated with dCCRT according to the N stage, GTVnd and TNM stage system, shown as **Figure 4**.

**Table 5:** Comparison of the prognostic performance of the TGTVndM and TNM staging systems.

Classification	Subgroups	Figure	Linear trend χ2	Likelihood ratio χ2	AIC
N stage	N0, N1, N2, N3	SA	5.06	11.68	1271.29
GTVnd stage	GTVnd<4.35cm^3^ GTVnd≥4.35cm^3^	SB	16.23	18.48	1260.23
TNM stage	I, II, III, IV	SC	25.77	20.69	1260.06
TGTVndM stage	I, II, III	3A	26.38	24.39	1255.07

## Discussion

Previous studies had reported the significant association between tumor volume and outcomes in EC patients treated with radical surgery (with or without neoadjuvant chemoradiotherapy) or definitive concurrent chemoradiotherapy (11–19). In our routine clinical practice, we found that EC patients with the same TNM stage and undergoing the same treatment might show considerably different clinical outcomes. We hypothesized that some patients with small tumors size and large lymph nodes metastases and those with large tumors size and small lymph nodes might had different prognosis. Indeed, the results of our study supported this hypothesis. In our study, we identified the GTVnd as an independent prognostic factor for OS, PFS and DMFS. Additionally, we identified a value of 4.35 as the optimal cut-off as defined by the RCS. The results showed that patients with GTVnd≥4.35 cm^3^ had shorter OS and PFS time and more often developed DM compared with GTVnd < 4.35 cm^3^. This is in agreement with previously reported findings. Sakanaka et al. reported 144 patients with thoracic ESCC who underwent definitive chemoradiotherapy with large metastatic lymph nodes were at high risk of DM in 2016 (20). In addition, Zhao.et al retrospectively reviewed 376 ESCC patients treated with definitive (chemo-) radiotherapy and concluded that bulky lymph nodes were associated statistically with distant failure and poorer OS (21). It was reported that lymph nodes metastases were more closely associated with systemic micro-metastases than primary tumor progression and a better lymph nodes response to neoadjuvant chemotherapy in patients with metastatic EC predicted a better survival and less lymphatic, distant metastases (20, 22, 23). Our study provides a scientific basis for the close correlation between lymph nodes metastases and tumor micro-metastases.

Then, we constructed a new prognostic model to divide ESCC patients into high-risk, intermediate-risk and low-risk groups using RPA method. The GTVnd was the first split and the second was clinical T stage in our RPA model. Specially, patients who were T1-4 and GTVnd≥4.35 cm^3^ were classified as high-risk group, T4 and GTVnd<4.35 cm^3^ as intermediate-risk group and T1-3 and GTVnd <4.35 as low-risk group. We found that patients with high-risk group exhibited shorter OS, PFS and DMFS compared with the other two groups and we also showed that the new staging system (TGTVnd) was superior to the traditional TNM staging system in predicting OS of ESCC patients treated with dCCRT. A previous study conducted by Chen et al. demonstrated that GTV and maximum diameter of metastatic lymph nodes (MDMLN) predicted survival of nonsurgical EC patients more accurately than the 8th edition of AJCC/UICC clinical staging system (15). Another study proposed a new nonsurgical staging system based on the gross tumor volume of the primary tumor and N to be better predict the outcome of ESCC patients (17). The majority of EC patients receiving dCCRT are diagnosed with advanced disease (stage III/IV), the survival of these patients varies widely and an effective staging system is desperately needed. The accuracy of CT for predicting the N staging was 78% in EC patients (24), which made inaccuracy of TNM staging to predict recurrence and prognosis to some extent. Therefore, we built a new risk staging system based on various combinations of T subgroups and GTVnd subgroups to better evaluate the outcomes of inoperable ESCC patients.

Moreover, the multivariate analysis also revealed that clinical T stage and gender were independent prognostic factors affecting OS for ESCC patients. A study from Taiwan analyzed 14,394 ESCC patients and indicated that sex and clinical T were independent prognostic factors for OS (25). This large sample cohort study supported the conclusions of our research. The multicenter studies at home and abroad demonstrated that men exhibited a worse prognosis than women in EC (26, 27). The reason for the discrepancy is not clear. Sex hormone might be one of the reasons to the difference in prognosis, and early preclinical research showed that estrogens significantly inhibited the growth of esophageal carcinoma cell (28). Another possible explanation could be differences in long term smoking history, heavy alcohol consumption and nutritional status between men and women (29, 30).

We found that the location of primary tumor was independently associated with recurrence in our study. It has been reported in previous studies that tumor location was an independent prognostic factor for PFS in EC patients (31, 32). Patients with cervical ESCC had the worse PFS rate (3-year PFS, 18.7%), compared with patients with thoracic ESCC (3-year PFS, 36.4%, P=0.002) in our study. Münch. et al. including 95 ESCC patients treated with either dCCRT or neoadjuvant chemoradiation followed by surgery reported that proximal tumor location was associated with short PFS (32), which was consistent with the results of ours. The location of the primary tumor relating to the mode of tumor recurrence might contribute to the difference (23, 32), to be specific, the local–regional recurrence rate was 72.2% for cervical ESCC while 43.9% for thoracic ESCC, showing a significant difference by the chi-squared test (P=0.01) in our study. Yang et al. reviewed 1220 thoracic ESCC patients who underwent complete resection to conclude that tumor location did not affect survival prognosis (33) and subsequent a study conducted by Shi et al. reported a decreased OS in pathological T2-3N0M0 ESCC patients with proximal tumor location (34). However, the different tumor locations have been used different treatment making comparisons across studies difficult. For example, dCCRT is the general recommendation for patients with cervical tumor location (35, 36) and surgery has been the standard treatment for thoracic EC (37). Overall, we should not neglect the tumor location as an important prognostic factor.

This study has several limitations, first, with the retrospective design based on case records, which may lead to bias in patient selection. Second, the difficulty in delineating metastatic lymph nodes due to partial lymph nodes conglomerated with primary tumor may cause certain error in a small number of patients. Finally, further studies with larger sample size and external validation are required to confirm our results. However, one strength of our study is that we investigated the effect of volumetric parameters on prognosis for patients with ESCC treated with dCCRT in the era of IMRT, which appears to be simple and accurate prognostic factors to be obtained. Moreover, we also constructed a prognostic risk group for inoperable ESCC patients, which might be a valid indicator for predicting the sensitivity to chemoradiotherapy.

## Conclusions

In conclusion, the present study showed that GTVnd was independently prognostically significant for OS, PFS and DMFS. The optimum cut-off point for GTVnd was 4.35 cm^3^ in predicting distant metastasis and death for ESCC patients treated with dCCRT. The new risk groups stratify these patients into I, II, and III subgroups to better assess the prognosis and guide the treatment. The results need validation from prospective randomized studies.

## Data availability statement

The original contributions presented in the study are included in the article/supplementary material. Further inquiries can be directed to the corresponding authors.

## Ethics statement

The study was approved by the Ethics Committee of Tianjin Medical University Cancer Institute and Hospital. The patients were not required to sign an informed consent form for this retrospective study.

## Author contributions

Conception and design: PW, QP, and WZ; Manuscript writing: YL; Data analysis and interpretation: YL and YQL; Data collection: HH, ZG, and KZ; Final approval of manuscript: All authors.

## Funding

This work was supported by Chinese National Key Research and Development Project (No. 2018YFC1315601) and National Natural Science Foundation of China (grants 81872462, 81972772 and 82073348).

## Conflict of interest

The authors declare that the research was conducted in the absence of any commercial or financial relationships that could be construed as a potential conflict of interest.

## Publisher’s note

All claims expressed in this article are solely those of the authors and do not necessarily represent those of their affiliated organizations, or those of the publisher, the editors and the reviewers. Any product that may be evaluated in this article, or claim that may be made by its manufacturer, is not guaranteed or endorsed by the publisher.

## Abbreviations

GTVnd, gross volume of metastatic lymph nodes; GTVp, gross volume of primary tumor; ESCC, esophageal squamous cell carcinoma; dCCRT, definitive concurrent chemoradiotherapy; IMRT, intensity-modulated radiation therapy; RCS, restricted cubic splines; RPA, recursive partitioning analysis; AIC, akaike information criterion; OS, overall survival; PFS, progression free survival; DMFS, distance metastasis free survival; LRFS, local recurrence-free survival; RRFS, regional recurrence-free survival; CTV, clinical target volume; PGTV, planning gross target volume; PTV, planning target volume.
